# Physical Wellbeing Monitoring Employing Non-Invasive Low-Cost and Low-Energy Sensor Socks

**DOI:** 10.3390/s18092822

**Published:** 2018-08-27

**Authors:** Laura García, Lorena Parra, Jose M. Jimenez, Jaime Lloret

**Affiliations:** Integrated Management Coastal Research Institute, Universitat Politècnica de València, C/ Paranimf nº 1, Grao de Gandía—Gandía, 46730 Valencia, Spain; laugarg2@teleco.upv.es (L.G.); loparbo@doctor.upv.es (L.P.); jojiher@dcom.upv.es (J.M.J.)

**Keywords:** wellbeing, low-energy, non-invasive, wearable

## Abstract

Determining and improving the wellbeing of people is one of the priorities of the OECD countries. Nowadays many sensors allow monitoring different parameters in regard to the wellbeing of people. These sensors can be deployed in smartphones, clothes or accessories like watches. Many studies have been performed on wearable devices that monitor certain aspects of the health of people, especially for specific diseases. In this paper, we propose a non-invasive low-cost and low-energy physical wellbeing monitoring system that provides a wellness score based on the obtained data. We present the architecture of the system and the disposition of the sensors on the sock. The algorithm of the system is presented as well. The wellness threshold evaluation module allows determining if the monitored parameter is within healthy ranges. The message forwarding module allows decreasing the energy consumption of the system by detecting the presence of alerts or changes in the data. Finally, a simulation was performed in order to determine the energy consumption of the system. Results show that our algorithm allows saving 44.9% of the initial energy in 10,000 min for healthy people.

## 1. Introduction

Due to the increase in life expectancy and aging of the population, especially in advanced countries, there is a need to monitor the health of people in a quick and efficient manner, particularly in the elderly, in order to provide them with a better wellbeing. As a way of monitoring wellbeing, some studies have begun to monitor the environment where people live [1], as it can affect both physical and psychological health. It is also very important to control the health of a part of the population formed by the chronically ill and physically disabled. The control and monitoring of health also has an important impact on the increase in economic costs, due to improper or inadequate medical treatment. Without this necessary control, the expense on medical treatments increases in patients who see their illnesses worsened by not receiving a rapid correction in their treatments.

Ensuring and improving the quality of Long-Term Care (LTC) services has become an important policy priority in all OECD countries, as presented in its report [2]. According to studies carried out by the OECD, it is expected that the proportion of people over 80 years of age will increase to 10% of the population by the year 2050. The measurement of LTC quality lags in comparison to the recent advances in medical care. The main challenge we face is how to control and obtain reliable patient data. The main current problem that is presented on the obtained data is that it is generally obtained by questionnaires. The information on these questionnaires is completed based on the answers granted by the users. The users provide subjective information, which may not adjust to reality. Another important challenge is to achieve a standardization of the collected data, in order to perform a clinical monitoring from different areas. To summarize, we can announce that the main objective to reach optimal levels of health care, with which an important part of the population made up of the elderly, physically disabled and chronically ill is served, is to implement data collection systems in an accurate, automatic, autonomous and standardized way.

Through the combination of non-invasive detection and the use of advanced computer technology, efficient, real and reliable monitoring of patients can be obtained. An optimal way to carry out the control of patients can be done by means of sensor monitoring. Using sensors allows getting information about both physical activity and vital signs, passively, non-invasively, discretely and if necessary continuously. The sensors can be integrated into mobile devices. These devices can be attached to the body or form part of our clothes. They are known as wearable devices [3]. The term wearables devices defines devices that generally resort to some wireless technology for the transmission of the data obtained by means of sensors, which record the personal information of the patients. Over the last few years, a large number of devices have proliferated to perform this function, among which we can highlight wristbands, watches, belts, shirts, caps, shoes, skin patches, etc. Among the usual functions performed by these devices to control body parameters are the monitoring of temperature, pulsations, heart and respiratory rhythms, blood pressure, etc. New, non-invasive, low-cost, low-energy sensors that can be used to monitor the physical well-being of people are presented every day. As a consequence of the increase of these devices, Wireless Body Area Networks (WBANs) are becoming more usual as a way of green communication [4], enabling wearable devices to communicate employing low energy and reliable transmission protocols [5]. Moreover, nowadays organizations like the FDA in the USA [6] or the NHS in the UK [7] are working to create a concrete legislation that regulates and certifies the devices that are considered “healthy” and safe.

In this paper, we propose a low-power consumption non-invasive physical wellbeing monitoring system incorporated in a sock. Our proposal incorporates a large number of parameters, such as body temperature, heart rate (HR), heart rate variability (HRV), oxygen saturation, the pressure exerted by feet, sweat and the level of activity. Low-cost sensors were considered for our proposal to be available to a large number of people. We also consider a minimum energy consumption for system, both for data collection and transmission. Therefore, we present an operation algorithm that optimizes data transmission in order to reduce energy consumption. Moreover, the algorithm is able to analyze the obtained data and alert the user in case of anomaly.

The rest of this paper is structured as follows: Section 2 presents some of the most relevant works related to monitoring human wellbeing. In Section 3 we present the materials and methods employed. The results and discussion are presented in Section 4. Finally, the conclusions are presented in Section 5.

## 2. Related Work

In this section related work from different papers on solutions for human wellbeing monitoring is presented. There are a lot of works and published papers focused at the design and deployment of multimedia sensors for e-health. Many of them base their operation on smartphones. Parra et al. [8] presented a collection of previously published works that use smartphones for e-health and ambient assisted living. Other authors as Lane et al. [9] present the design, implementation and evaluation of an automated wellbeing app named BeWell and demonstrate its feasibility in monitoring multi-dimensional wellbeing. The authors affirm that with this tool can detect personal wellbeing, related to a generic way, appreciating any variation.

Mund el al. [10] have presented an unobtrusive and wearable, multiparameter ambulatory physiologic monitoring system for space and terrestrial applications that they named LifeGuard. The monitored parameters could be recorded digitally with high fidelity over a 9-h period. The system allowed transferring the data recorded from a cell phone to a base station using Bluetooth, or stored it in 32 MB of on-board flash memory and downloaded it to a personal computer using a serial port. In addition, the buzzer alarm of the cell phone can be activated whenever abnormalities are observed.

Authors such as Miramontes et al. [11] have presented a platform called PlaIMoS which consists of wearable sensors, a fixed measurement station, a network infrastructure that employs IEEE 802.15.4 and IEEE 802.11, a server to analyze all the collected information and apps for different operating systems to provide real-time measurements. They have developed an architecture to record and report electrocardiogram and heart rate data. It also monitors parameters associated with chronic respiratory illnesses. Nowadays, we can find devices that are capable of monitoring the heart rate using single-lead electrocardiogram (ECG). For example, the device provided by Zio Patch [12] is an adhesive patch. Its manufacturers say it allows a clear monitoring of the heart rate of the patient without losing critical information for 14 days. In their specifications, they say that with Zio Patch it is possible to avoid critical knowledge gaps from interrupted data. We can simply choose the best time frame for the patient and use their comprehensive report to make a confident diagnosis. However, while wearing the monitor, the providers say that the following tests or treatments are not recommended: magnetic field(s), neuromuscular stimulators and external cardioversion/defibrillation.

Henry et al. [13] have developed a non-invasive, wireless platform to continuously monitor cardiac output (CO), stroke volume (SV) and a new parameter that they call Cardiovascular Reserve Index (CRI) to estimate acute blood loss volume and predict cardiovascular decompensation well in advance of clinically significant changes in currently available vital signs. It is known that, in several situations, hemorrhagic shock induced by a traumatic injury is the main cause of mortality. The authors state that conventional vital signs, such as heart rate and blood pressure, are generally nonspecific and slow to change until the acute blood loss volume nears 25%–30% of total blood volume. The authors say that the indicators they have studied are superior to know blood loss volume and fluid resuscitation needs and they have demonstrated their value. They implemented a small noninvasive body-worn device wirelessly connected to a central monitoring system.

Authors like Sahoo et al. [14] have presented a continuous and non-invasive cardiac health monitoring system using unobtrusive sensors which aims to provide a feasible and low-cost alternative to foresee possible cardiac anomalies in an early stage. They used a novel low-cost, non-invasive seismocardiogram (SCG) signal along with ECG signals which are jointly investigated for a robust cardiac health monitoring. A combined analysis of ECG and SCG is carried out by designing a Naïve Bayes conditional probability model. Experiments on Institutional Review Board (IRB) approved licensed ECG/SCG signals acquired from real subjects containing 12,000 cardiac cycles showed that the proposed feature point delineation mechanisms and abnormal morphology detection methods consistently perform well and give promising results. In addition, their experimental results show that their combined analysis provide more reliable cardiac health monitoring compared to the standalone use of ECG and SCG.

Other authors such as Trung et al. [15] have presented a set of transparent and stretchable (TS) gated sensors, as well as the integrated platform of the TS gated sensor with a transparent and stretchable strain sensor. The authors say that their sensors show great potential for application to wearable skin electronics for recognition of human activity. In addition, the authors say that the temperature sensor can be adapted to both objects and the human body to control the temperature.

Steinhubl et al. [16] presented a sensor for the treatment of ebola, which was developed by the STAMP2 consortium and sponsored by the United States Agency for International Development (USAID). The sensor monitors multiple vital signs continuously and remotely. It is similar to a Band-aid, so it is portable, and transmits the information monitored wirelessly to those responsible for medical care that are far from the observation areas.

Other authors have studied the activities of daily life. For example, Ozemek et al. [17] presented the triaxial accelerometer GT3X+. According to the authors, one can measure activity in the vertical, horizontal from right to left and horizontal directions from front to back planes. They place their device on the hip, on the wrist and on the ankle. They performed tests on forty individuals and their results suggest that they are reliable. Other authors such as Carlijin et al. [18] presented the development of an accelerometer and a portable unit for data processing with which they evaluate physical activity. They evaluated the presented system in a group of 13 individuals. They found deficiencies in their system due to the fact it did not obtain adequate precision when measuring sedentary activities and the impossibility of registering static exercise.

Ghamari et al. [19] proposed a model to represent a photoplethysmography signal (PPG) as the sum of two Gaussian functions, with the aim of analyzing the Heart Rate Variability (HRV). The authors designed a bracelet-type PPG device with which the arterial pulse is monitored from the wrist. The bracelet incorporates a Bluetooth module to transfer the observed data, in addition to an optical sensor, a microcontroller and a unit to amplify and filter the analog signal of the PPG. At the end of the work they present a procedure to verify the HRV signal during a sedentary activity.

Low et al. [20] performed a study of the accuracy and reliability of two pairs of Footscan pressure templates (500 Hz, RSscan, Paal, Belgium). Measurements were taken with eight participants. Later they compared the measurements obtained with those of a force plate (500 Hz AMTI, Watertown, MA, USA). In their final results they say that, although their measurements were reliable, the data of the template were lower. They advise that an adequate calibration of the templates be carried out before being used.

Finally, Paulo Rodrigues et al. presented in [21] a sock for biometric monitoring. Their patented device measures biometric parameters like temperature, pressure and heart rate and employs conductive yarns to forward the information gathered from sensors to the monitoring device. These biometric parameters can be employed to determine the physical effort performed by the user. The sock incorporates on the bottom a 10 mm thick gel layer and a GPS tracker.

The aforementioned works show different sensors and systems can be used for health and physical wellbeing monitoring. However, the described systems only consider some aspects of the entire system like the calibration of the sensor, its location or they are focused on just a few parameters, some of them focusing on only one parameter such as heart rate or foot pressure. As far as we know, there is no other paper that presents a proposal for health monitoring and focuses their efforts on the system description, including the packet exchange, the operation algorithm and the inclusion of energy saving algorithms considering the gathered values. This is the gap that we are going to cover with this publication, where we present the operation algorithm of a system for physical wellbeing monitoring and its energy saving by using smart algorithms.

## 3. Materials and Methods

In this section, the selected wellness monitoring parameters are discussed. Moreover, the architecture of the system is presented as well as the message exchange between the elements of the architecture and the algorithm of the performance of our proposal.

### 3.1. Wellness Parameters

The wellness parameters selected for this proposal are depicted in this subsection. Sensors allow obtaining a wide variety of types of information. Regarding human wellbeing, we have selected seven parameters in order to monitor the current state of a person. The proposed parameters are body temperature, heart rate, heart rate variability, oxygen saturation, the pressure exerted by feet and the level of activity. The proposed system is intended to provide the user with an assessment of their wellbeing. It is not intended to be a system for medical diagnosis and, in case of alarm readings, the user should visit a medical clinic.

Body temperature is one of the parameters that most define the current wellbeing of a person. It is very important for the user to stay within a healthy temperature value range. Body temperature can vary throughout the day with fluctuations usually no higher than 1 °C. High temperatures o or fever can be caused by infections, inflammations or hyperthermia. Low body temperatures can be caused by hypothermia. Normal body temperatures can differ in accordance to the age of the person. Thus, older adults may present hypothermia from 35 °C downwards while hypothermia in children may be considered from 36 °C [22]. Nowadays there are a wide variety of sensors that allow monitoring body temperature. Many of them are low cost sensors like the LilyPad Temperature Sensor for wearable solutions [23] that allow monitoring body temperature satisfactorily enough for a solution with the characteristics of our proposal. The datasheet of the sensor that provides its accuracy can be accessed in [24].

Heart rate is another one of the three parameters of our system that are crucial to human wellbeing. Physical exercise, medication or emotions are some of the factors that can affect HR. Very low heart rate or frequent high HR can be alarming [25]. Photolethysmography sensors are widely employed for HR monitoring. We propose the usage of pulse sensors such as the Pulse Sensor developed by SparkFun [26]. Its datasheet can be accessed in [27].

Associated with HR is heart rate variability also known as rMSSD which is measured in ms. HRV is the time between pulse beats. Higher HRV values are considered more beneficial. Lower HRV values indicate equidistant heart beats and are associated with stress [28]. Pulse sensors as the one proposed for HR monitoring and ECG sensors can be employed for monitoring this parameter.

Oxygen saturation is the last parameter measured by this system that determines if a person is healthy. This parameter is measured in percentages. Normal values vary from 95% to 100%. Values below 94 can be alarming and require the incorporation of oxygen masks to ensure correct breathing and values below 90 are considered very low. Low oxygen saturation values are called hypoxemia. Red and infrared light is employed to perform the oximetry in the SparkFun MAX30105 [29] sensor that we propose to use for our system. Its datasheet is available in [30].

Pressure sensors can be employed for monitoring correct posture during walking. Several studies have been performed on smart insoles that allow monitoring the pressure that feet make on the insoles as well as determining the habits of the user when walking, such as being pronator or supinator [20]. These insoles are comprised of force sensitive sensors deployed all over the insole to obtain data from varied points in the surface of the feet. We propose the FSR (Force Sensitive Resistor) 0.5” pressure sensor [31]. Its datasheet can be accessed on [32].

Accelerometers are often employed in e-health to monitor physical activity. Measures can be performed in three axes and patterns are identified in order to discern among several activities like walking, running, lying down or stand in one place [33]. Four our proposed sensor sock, we consider the usage of the ADXL335 triple axis accelerometer [34]. Its datasheet can be accessed on [35].

Finally, sweat can be measured employing galvanic skin sensors which measures the electrical conductance of the skin. We propose the usage of the Grove-GSR sensor [36]. Its specifications are presented in [37]. Emotional state, body temperature or ambient temperature are some of the causes of body sweat.

Due to the need of a node that can be incorporated in clothing pieces, in this case, the ankle part of a sock, we propose the use of wearable nodes such as the Arduino Lilypad as employed in our previous work [38]. In order to allow the transmission of the monitored data, a wireless module is required. The Wifi Bee ESP V1.0 board [39] allows providing WiFi connectivity to the LilyPad Arduino board. Most of the aforementioned sensors are intended to be used in contact to the skin. The appropriate measures to protect the sensors and connections to the effects of sweat would be taken. Moreover, some of the aforementioned sensors are intended for textile applications and there are textile conductors available in order to incorporate connections to pieces of clothing.

In this paper, we also propose a points-based score, similar to that proposed on our previous work [28], in order to quickly assess the wellness of the user and as a way of presenting the user with a simple first assessment. Results on these parameters can be further consulted without considering the obtained score. Table 1 presents the parameters with their respective ranges and the points assigned to each of them. The minimum score that the user can receive is 0. Although the system may use negative values for calculation purposes, if the resulting points are negative, the score will be 0. The score range that can be received is depicted in Table 2. This score is a guide to the wellness state of the user and should not be employed as a QoL (Quality of Life) score.

### 3.2. System Description

**Figure 1:**
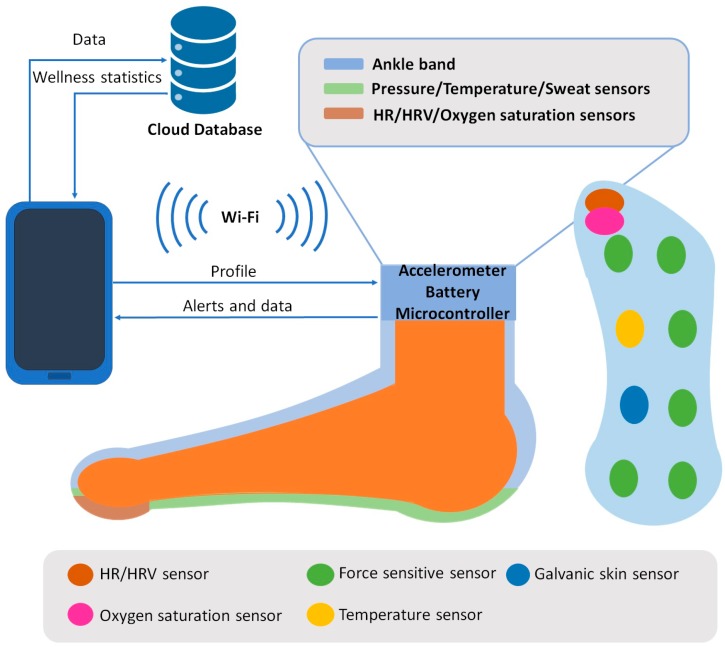
Architecture of the proposed system.

In this subsection, the architecture of the system is presented. The message exchange among the elements of the system is presented as well. Moreover, the algorithms of our proposed wellbeing monitoring system are presented. The architecture of the system is presented in Figure 1.

Pressure, temperature HR, HRV, sweat and oxygen saturation sensors are deployed all over the sole of the sensing sock. The locations of the sensors have been selected considering works like [20] and [21] and the needs of each sensor to capture the data correctly. Thus, pressure sensors are deployed at the parts of the insole were the foot applies more pressure. Temperature and oxygen saturation sensors are located below the thumb in order to obtain the best reading. Works like [21] and [40] have been considered to assess that these parameters can be measured on feet. The temperature sensor and the sweat sensor are located on the part of the insole where they cannot interfere with other sensors, considering as well that measures can be performed successfully. Finally, the accelerometer is located at the ankle band as activity can be monitored similarly to wrist bands. The information of these sensors is gathered by the node located on the ankle band that comprises the open extreme of the sock. This ankle band also has an accelerometer and the battery of the system. The gathered data is processed locally on the node applying the proposed algorithms. The node and the smartphone stablish communication through Wi-Fi. The profile of the user is forwarded to the node so the ranges for each wellness parameter are adequate. This way, the monitored data of athletes and non-athletes are processed in accordance to their habitual HR and HRV. When data is processed, it is forwarded to the smartphone, where the user is able to access the data and the wellness score provided by the system. Alerts are forwarded to the smartphone as well. These alerts occur when the measured vital parameters exceed healthy values. A cloud database receives the data as well, which at the same time, can be employed to perform statistics on the results measured by the system. These statistics can be forwarded to the smartphone for the user to access them.

**Figure 2:**
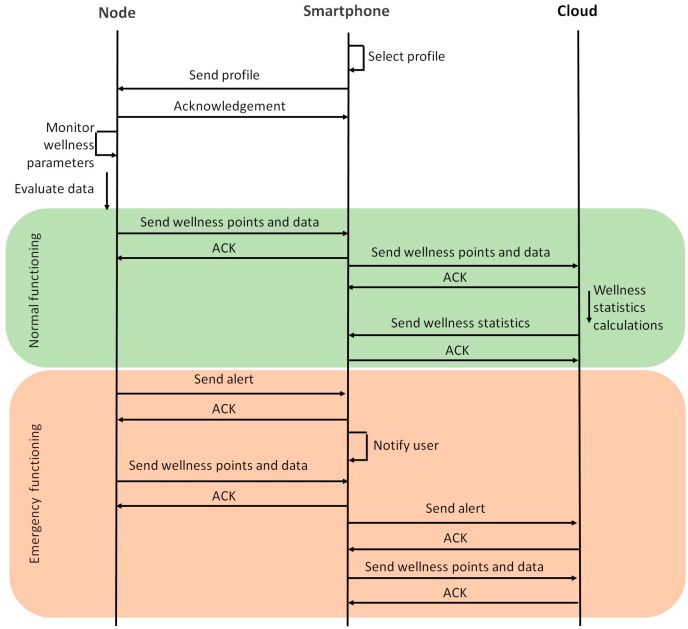
Message exchange between the elements of the system.

The message exchange of the system is presented in Figure 2. The smartphone selects the profile of the user and then, it is forwarded to the node located in the ankle band of the sock. As TCP is employed to forward the data, an acknowledgment is forwarded to the smartphone to indicate that the message has been received successfully. The messages involved in the handshake process have been omitted in the figure. Then, the node starts monitoring the wellness parameters and evaluating and processing the measures. If the system is performing normally, the data and wellness points are forwarded each 30 min if changes have been registered in HR, HRV, temperature, oxygen saturation or sweat parameters. This time interval has been selected after performing some previous measures and consulting documentation on the variation of the parameters considered on this paper such as [41,42]. The accelerometer and pressure sensors may vary more frequently, whereas body temperature and oxygen saturation are parameters that remain constant for larger periods of time. The importance of each parameter to the wellbeing of the user was another factor considered in this decision. The smartphone then forwards an ACK to the node. The data and wellness points are also forwarded to the cloud in order to be stored and analyzed for statistics calculations. Then, these statistics are transmitted to the smartphone of the user. If the performance of the system is in emergency mode, the node sends an alert to the smartphone in order to notify the user of an anomaly on temperature, HR, HRV or oxygen saturation parameters. Then, the node forwards all the data from the last 30 min so the user can access the information that has caused the alarm. Then, both the alarm and the data and wellness points are forwarded to the cloud, so they can be registered.

**Figure 3:**
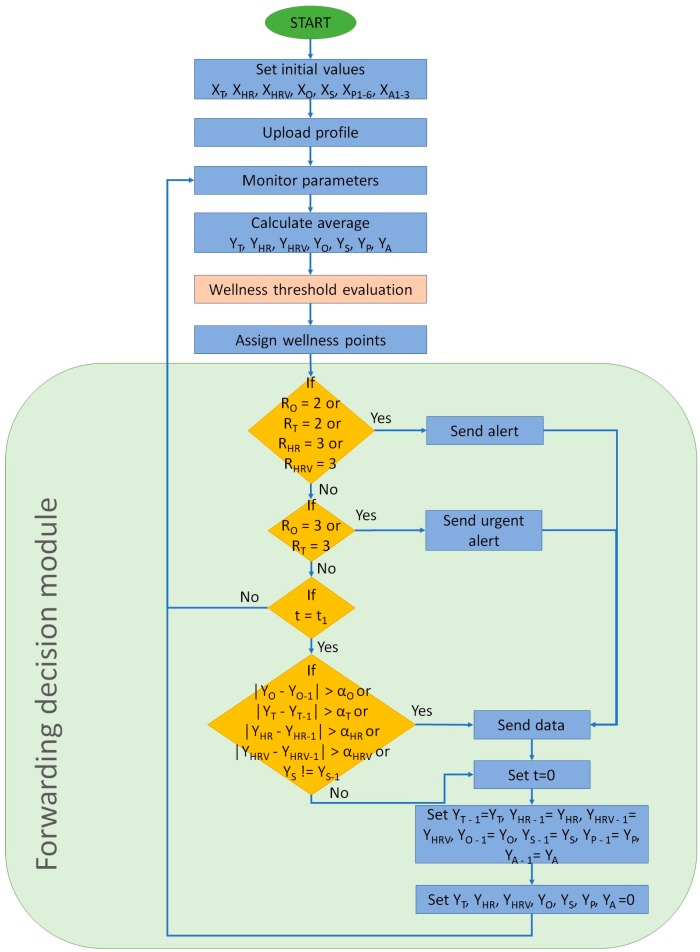
Algorithm of the performance of the system.

**Figure 4:**
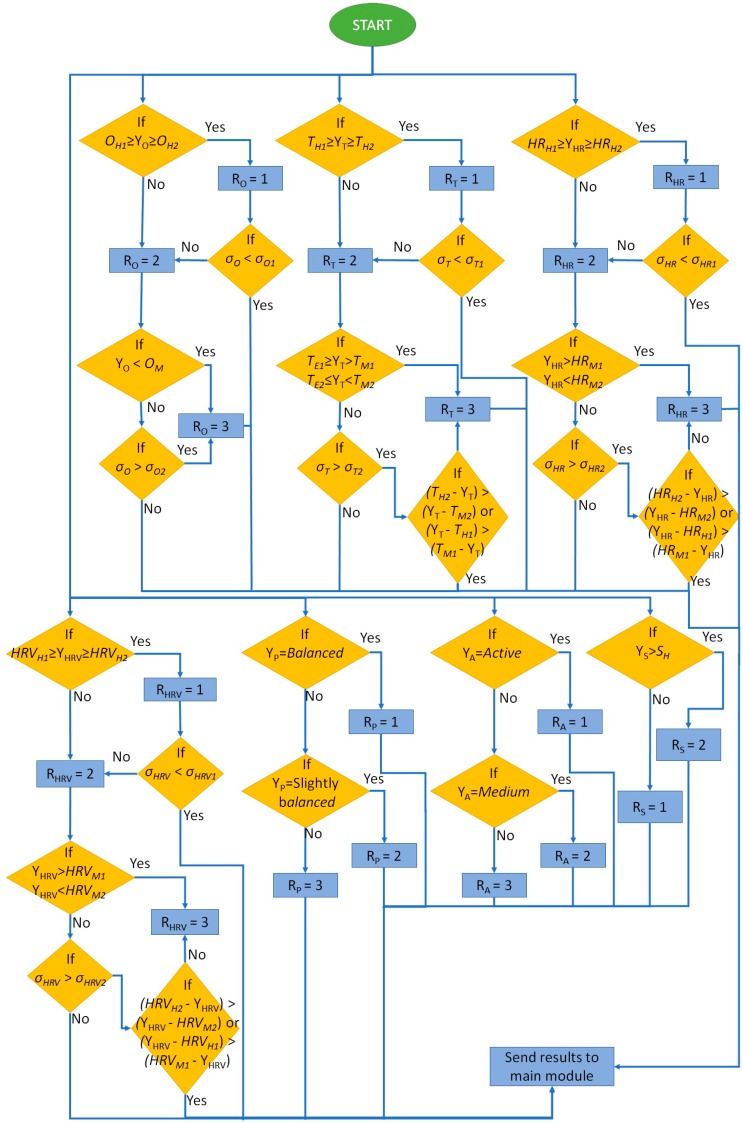
Algorithm for the wellness range evaluation.

Figure 3 presents the main algorithm of the performance of the system. It includes the message forwarding decision module. Firstly, initial values are set to 0. Table 3 presents the description of the parameters employed in both algorithms. Then, the profile of the user is uploaded. Athletes may obtain different readings from people who exercise less. In this paper, the normal profile is employed. Then, wellness parameters are monitored. The average of these parameters is calculated and forwarded to the wellness range evaluation module. This module is presented in Figure 4. Afterwards, points are assigned regarding the results obtained from the wellness range evaluation module. Following, the algorithm enters the message forwarding decision module, which is the part of the algorithm that allows reducing the energy consumption of the system. The number of transmissions is determined by the changes of the monitored parameters and the alerts received from the wellness range evaluation module. If either oxygen saturation or body temperature have received the value 2 or, HR or HRV received the value 3, an alert and the data is forwarded to the user. If the aforementioned values have not been obtained, the system evaluates if either oxygen saturation or body temperature have received the value 3 and, therefore, an urgent alert and the data is forwarded to the user. If none of the aforementioned cases are true, the system waits until t1 min have passed. Then, it evaluates if there has been any significant change in the wellness parameters that most affect the wellness state of the user. If there have not been any changes, no data is forwarded, and the system continues its monitoring activities. If there are changes, all data is forwarded before continuing with the monitoring activities. Table 4 presents the description and values of the parameters employed in both Figure 3 and Figure 4.

The algorithm for the wellness range evaluation module is presented in Figure 4. Each wellness parameter is evaluated in accordance to the ranges specified in Table 1. Firstly, if the average of the measured values for the oxygen saturation parameter is within the healthy range, the value 1 is assigned. However, in order to detect values from other ranges, the standard deviation is evaluated. When the maximum standard deviation that can be obtained with the values within the healthy range is surpassed, the range value, RO in this case, receives the value 2. This way, each range is identified. The same happens for the middle and extreme ranges. A conditional evaluates the average of the measured parameters in order to determine in which range it is. If the middle range is selected, the standard deviation is compared to the maximum one that can be obtained in the middle range. If it is surpassed, the value 3 is assigned, indicating an extreme alert. Temperature, HR and HRV are very similar to the methodology employed for oxygen saturation. However, if the average is within the middle range but the maximum standard deviation is surpassed, a conditional evaluates if the measured values that belong to other range are from the healthy range or the extreme range. In order to do that, the subtraction between the average and the values that define the ranges is performed. The lowest result indicates the closest range. If the user is exercising, more alerts concerning HR could be forwarded. However, it is an expected behavior of the system and the user should not be preoccupied. Moreover, HR alone is not a trigger of urgent alerts. Pressure and the activity measured by the accelerometer is determined by the amount of movement. These parameters vary constantly and, due to difference of importance to the aforementioned wellness parameters if the measures reach the extreme range, pressure and activity are not considered for forwarding alerts to the user. Finally, sweat is a boolean value and only its existence or absence is considered. Therefore, the value would be true when the user is sweating and false when the user is not sweating. It is also not considered for alerts. Lastly, the results are sent to the main algorithm.

**Figure 5:**
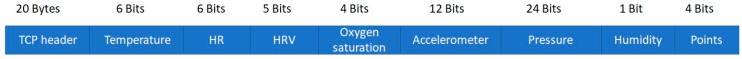
Message format of our system.

For our system we propose a modification of the TCP protocol. The message for data forwarding is presented in Figure 5. It is comprised of the TCP header, followed by the temperature field which has the size of 6 bits. It is then followed by the HR and HRV fields with 6 and 5 bits respectively. Oxygen saturation is the next field with 4 bits. The last fields related to the wellness parameters are accelerometer, pressure and humidity with 12, 24 and 1 bit, respectively. Finally, the last field is the wellness points provided by the system which has a size of 4 bits. The ACK message only employs the TCP header.

## 4. Results and Discussion

**Figure 6:**
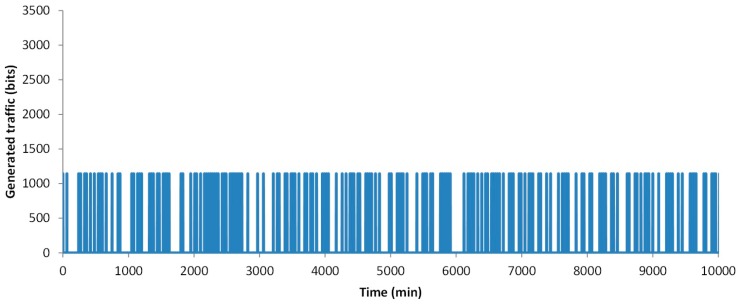
Generated traffic by a node used by a healthy person.

**Figure 7:**
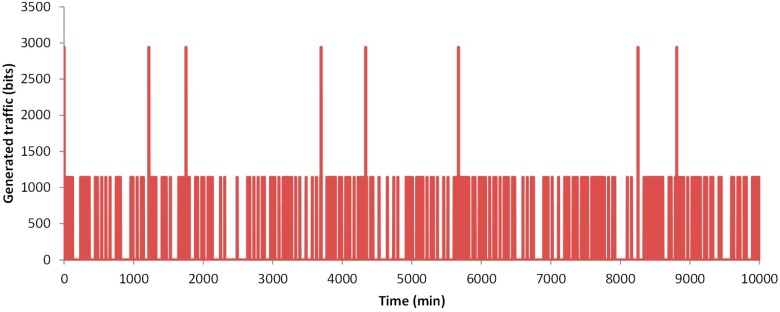
Generated traffic by a node used by a person with cardiac affection.

**Figure 8:**
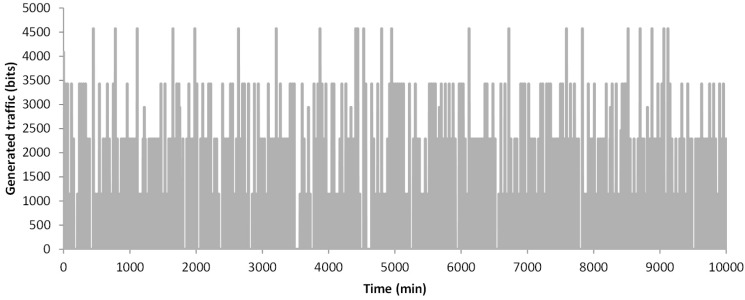
Generated traffic by a 4-member family.

In this section, we are going to represent the results of the simulated data. First, we are going to show the generated traffic through our system. Then, the energy issues are presented. The generated traffic in our system depends on two factors. The first one is the generation of an alarm message, which can appear in any minute of the simulation. The second one, the need of sending data because a change is detected, and the new data must be sent. This can happen every 30 min when the algorithm analyses the gathered data. For this simulation, the traffic generated by the data message as stated in Section 3.2 is considered, including the handshake process and the acknowledgements. The results of the generated traffic include the generated traffic by a node that gathers data from a healthy person, a node that gathers data from a person with a cardiac affection and the generated traffic by a family, with three healthy people and one person with a cardiac affection. In the case of a healthy person, after 10,000 min of simulation, no alarm was generated (see Figure 6). Nevertheless, on many occasions, the data from measured parameters has changed and it needs to forward the new values. During the 10,000 min, in 162 occasions a message exchange to notify changes in the measured parameters was required. The total generated traffic during the entire simulation between the node and the AP is 185,004 bits. However, during most of the simulation, no traffic was generated by the node. Figure 7 shows the generated traffic by a node used by a person with cardiac affection. This user will have more possibilities to generate an alarm. A total of eight alarms where generated. In addition, in 201 occasions the algorithm decides to send the information. The total generated traffic is 243,926 bits during the entire simulation. Finally, we present the generated traffic by our system in a house where four members are living together and using the proposed system, where one member of the family has a cardiac affection and the rest are healthy people (see Figure 8). During the entire simulation, a total of 727 events, where the information must be sent, were registered. The total generated traffic was 844,138 bits, with an average traffic of 84 bits/min (1.4 bits/s). The maximum generated traffic was 4568 bits in one minute of simulation.

**Figure 9:**
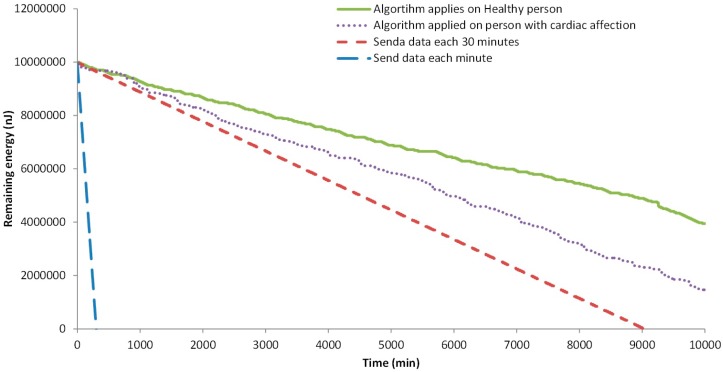
Remaining energy in each node.

Now we are going to show the results of the energy use when transmitting the data according to our algorithm or when the node transmits data each 1 min or 30 min, without considering any alarm or the changes in the evaluated parameters. The energy consumption simulations were performed employing the same methodology as in [1], which is based on [43] and [44]. Again, two different situations are considered when we apply the algorithm, the healthy person and the data from a person with cardiac affection. For these simulations the normal profile is employed. The athlete profile implies a change in the employed thresholds of the algorithm for the Heart Rate parameter according to their usual baseline heart rate. Therefore, alerts would happen in a similar manner to those in people with normal activity. In Figure 9 the results of our simulations are presented.

When no algorithm is used, and the information is sent each minute, the available energy for data transmission ends after 306 min. The node that sends the data every 30 min falls after 9035 min of simulation. Finally, the nodes that employ our algorithm, after 10,000 min still have energy. The node that is processing the data from the healthy person has 44.9% of the initial energy, 4,497,432 nJ. The node that is processing the data from the person with the cardiac affection has less remaining energy because more data was sent in the alarms and more events are triggered. The remaining energy is 2,149,464 nJ, the 21.5% of the initial energy.

**Figure 10:**
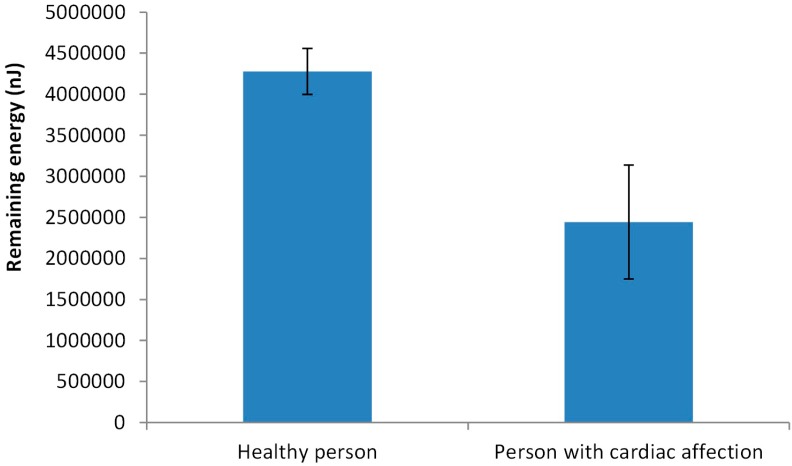
Remaining energy in each node after 10,000 min.

In order to determine with more detail the consumed energy by the generated traffic (sending and receiving data), the following analyses were done. First, the remaining energy after 10,000 min in 20 simulations is considered. Then, we analyze the time of node loss its energy in 20 different simulations. Both nodes, with data from the healthy person and with data from a person with cardiac affection, are tested. The results of the remaining energy after 10,000 min of simulation are shown in Figure 10. We can see that the average remaining energy in the node from a healthy person is 4,277,526 nJ (42.8% of the initial energy). The minimum and maximum remaining energy is 47.9% and 37.5% of the initial energy for data transmission. The standard deviation of the results obtained from the simulations is 281,497 nJ. Regarding the node that sends the data from the person with cardiac affection, the average remaining energy after 10,000 min is 2,442,992 nJ, the 24.4% of the initial energy. The calculated standard deviation is 693,088 nJ. The maximum and minimum remaining energy is 37.8% and 11.3% of the initial energy. We can see that there is a higher variation in the node with data from the person with the cardiac affection.

**Figure 11:**
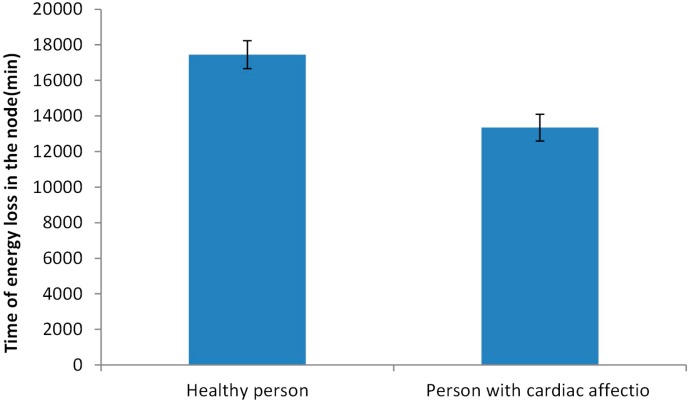
Lifetime of the available energy for data transmission.

Finally, the data related to the moment when the node consumes the energy available for data transmission is shown in Figure 11. Again, the two different situations are considered. As it is expected the node that sends data from healthy persons have a longer lifetime. The average lifetime of the nodes is 17,439 min from the node with data from the healthy person and 13,343 for a node with the data from a person with cardiac affection. In this case, the standard deviation of the lifetime of nodes in the simulation is similar, 787 min for the node with data from the healthy person and 756 for the other. The maximum and minimum lifetime for the node that sends the data from the healthy person is 18,690 min and 15,570 min. For the other node, the values are 15,480 min and 12,320 min.

Thus, our simulated results show that our algorithm supposes a reduction in the generated traffic and in the energy consumed, compared with sending all the data every 30 min. However, the level of improvement in energy saving depends on the personal condition of the user. If the user has any type of affection and it generates several alarms, the energy saving is reduced. In addition, as our algorithm reduces the generated traffic, the impact on the available bandwidth in the domestic environments is not significant. Considering that the maximum generated traffic by our system is less than 5000 bits, it is possible to use this system by everyone in the house without having negative effects on the internet connectivity of other devices. Other proposals for biomedical parameter monitoring such as the one presented by Nia et al. in [45] calculate energy consumption by utilizing several formulas. Their proposal considers eight different parameters and the battery lifetime for each sensor employing a 3 V coin cell battery. However, authors consider energy consumption for each sensor individually obtaining a maximum of 192.9 days of battery life for the heart rate sensor, 192.86 days for the accelerometer and 10,125.69 days for the oxygen saturation sensor. Nonetheless, comparing the energy consumption of health monitoring devices present several challenges due to differences in the number of sensors employed in the system, the frequency of data transmission, the amount of data to be transmitted, the selected battery and the methodology employed for measuring energy consumption.

**Figure 12:**
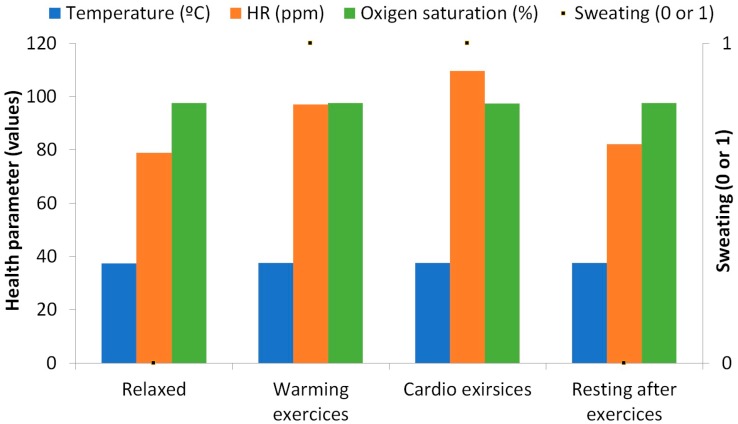
Wellness parameters measurements from preliminary tests of the prototype.

Even though the main objective of the present paper is to show the results in terms of energy saving of our proposed system, some real test with the sensors were done. In Figure 12 we present the data gathered by some of the sensors employed in the aforementioned prototype. The data related to the pressure sensors are not employed in this graphic because the sensors are not ready for the real test. The data was gathered from a 29 years old female during her daily exercise routine. Five different repetitions are taken for each variable and the data shown in Figure 12 is the mean of the gathered values. The first data is gathered before starting the exercises during a resting period. The data from temperature was constant in this case with a mean value of 37.4 °C. The HR has a variation of less than 5 ppm with a mean value of 78.8 ppm. The oxygen saturation has a mean value of 97.6 and no sweating is detected at this time. The second set of data belong to the warming exercises. In this case, the most relevant variation is related to the HR that increases to 97 ppm. Moreover, it has a greater variation (minimum of 92 ppm and a maximum of 104 ppm). The temperature and the saturation are almost the same as that in the first period. The sweating sensor detects sweating in 4 cases. Next, the sensors gather data during the cardio exercises. The HR increases to 109 ppm, with a maximum value of 116 ppm. The temperature increases to 37.6 and the sweating sensors detect sweating in five cases. Finally, more data is gathered during the resting period after the exercises, this data follows the same pattern that the data from the first case.

## 5. Conclusions

In this paper, a non-intrusive low-cost low-energy sensor sock has been proposed. The system is able to monitor the wellbeing of the user and provide a score in regard to the values obtained for each parameter. A wide variety of parameters have been considered in the system being HR, HRV, oxygen saturation, body temperature, pressure on the soles of the feet, physical activity and sweat. The algorithm that details the performance of the system has been shown. The wellness range evaluation module allows determining the state of the user for each parameter and assigns a reference value in order to set up the alarm in case it is necessary. The forwarding decision module of the algorithm allows determining whether the data should be forwarded or not. In order to do so, the system determines if an alarm has been activated or if there have been any significant changes to the monitored data. Simulations have been performed considering healthy and ill people as well as for one, two and four people. Results show a 44.9% of the energy remains after 10,000 min for a healthy person and 21.5% in the case of the ill person.

As future work, we will implement and test the prototype with users of various characteristics. Moreover, we will combine the wellbeing monitoring system proposed in this paper with our previous domestic environment monitoring system [1] in order to perform a complete monitorization of the parameters that may affect human wellbeing, both physical and external. We will consider as well other methods of wellbeing monitoring for both domestic and urban environments.

## Figures and Tables

**Table 1:** Wellness assessment score distribution of points.

Parameter	Healthy Range			Medium Range		Extreme Range	
	Low	High	Points	Range	Points	Range	Points
HR	50	100	3	±20	2	−30/+50	−100
HRV	50	100	3	±20	2	±50	1
Temperature	35	37	3	±2	0	−95	−100
Oxygen saturation	95	100	3	−2	−10	±8	−100
Pressure	Balanced		3	Slightly unbalanced	2	Unbalanced	1
Activity	Active		3	Medium	2	Sedentary	1
Sweat	0	0	1	1	0	-	-

**Table 2:** Ranges of the wellness evaluation points and their description.

**Points Range.**	0–4	5–9	10–13	14–16
**Description**	Extremely bad	Bad	Good	Excellent

**Table 3:** Description of the parameters employed in the main algorithm and the algorithm for wellness range evaluation.

Parameter	Description	Parameter	Description
Y_O_	Oxygen saturation average	R_A_	Current range of accelerometer
Y_T_	Body temperature average	σ_O_	Standard deviation of oxygen saturation
Y_HR_	HR average	σ_T_	Standard deviation of body temperature
Y_HRV_	HRV average	σ_HR_	Standard deviation of HR
Y_S_	Sweat average	σ_HRV_	Standard deviation of HRV
Y_P_	Pressure average	Y_O-1_	Previous oxygen saturation average
Y_A_	Accelerometer average	Y_T-1_	Previous body temperature average
R_O_	Current range of oxygen saturation	Y_HR-1_	Previous HR average
R_T_	Current range of body temperature	Y_HRV-1_	Previous HRV average
R_HR_	Current range of HR	Y_S-1_	Previous sweat average
R_HRV_	Current range of HRV	Y_P-1_	Previous pressure average
R_S_	Current range of sweat parameter	Y_A-1_	Previous accelerometer average
R_P_	Current range of pressure		

**Table 4:** Parameters and values employed in both the main and the wellness range evaluation algorithms.

Parameter	Description	Value	Parameter	Description	Value
O_H1_	Oxygen saturation healthy range max. value	100	T_E2_	Body temperature extreme range min. value	30
O_H2_	Oxygen saturation healthy range min. value	95	σ_T1_	Max. value for standard deviation of the healthy range of body temperatures	1
O_M_	Oxygen saturation medium range value	93	σ_T2_	Max. value for standard deviation of the middle range of body temperature	0.95
σ_O1_	Max. value for standard deviation of the healthy range of oxygen saturation	2.5	HRV_H1_	HRV healthy range max. value	100
σ_O2_	Max. value for standard deviation of the middle range of oxygen saturation	0.5	HRV_H2_	HRV healthy range min. value	50
HR_H1_	HR healthy range max. value	100	HRV_M1_	HRV medium range max. value	120
HR_H2_	HR healthy range min. value	50	HRV_M2_	HRV medium range min. value	30
HR_M1_	HR medium range max. value	120	σ_HRV1_	Max. value for standard deviation of the healthy range of HRV	25
HR_M2_	HR medium range min. value	30	σ_HRV2_	Max. value for standard deviation of the middle range of HRV	9.5
σ_HR1_	Max. value for standard deviation of the healthy HR range	25	S_H_	Value for sweat average	0.5
σ_HR2_	Max. value for standard deviation of the middle range of HR	9.5	t_1_	Time for forwarding data	30 min
TH1	Body temperature healthy range max. value	37	αO	Oxygen saturation threshold	4
TH2	Body temperature healthy range min. value	35	αT	Body temperature saturation threshold	0.4
TM1	Body temperature medium range max. value	39	αHR	HR threshold	14
TM2	Body temperature medium range min. value	33	αHRV	HRV threshold	14
TE1	Body temperature extreme range max. value	43			

## References

[1] García L., Parra L., Romero O., Lloret J. (2017). System for monitoring the wellness state of people in domestic environments employing emoticon-based HCI. J. Supercomput..

[2] OECD Health Policy Studies “A Good Life in Old Age? MONITORING AND IMPROVING QUALITY IN LONG-TERM CARE”. http://www.oecd.org/health/health-systems/good-life-in-old-age.htm.

[3] Pantelopoulos A., Bourbakis N.G. (2009). A Survey on Wearable Sensor-Based Systems for Health Monitoring and Prognosis. IEEE Trans. Syst. Man Cybern. C Appl. Rev..

[4] Khodabandeh H., Ayatollahitafti V., Taghizadeh M.S. (2017). Link aware and Energy efficient Routing Algorithm in Wireless Body Area Networks. Netw. Protoc. Algorithms.

[5] Masud F., Abdullah A.H., Abdul-Salaam G., Ishfaq M.K. (2018). Emergency Traffic MAC Protocols in Wireless Body Area Networks. AD HOC Sens. Wirel. Netw..

[6] U.S. FOOD & DRUG ADMINISTRATION. https://www.fda.gov/.

[7] NHS. https://www.nhs.uk/pages/home.aspx.

[8] Parra L., Sendra S., Jimenez J.M., Lloret J. (2016). Multimedia sensors embedded in smartphones for ambient assisted living and e-health. Multi. Tools Appl..

[9] Lane N., Rabbi M., Lin M., Campbell A.T. BeWell: A Smartphone Application to Monitor, Model and Promote Wellbeing. Proceedings of the 5th International ICST Conference on Pervasive Computing Technologies for Healthcare.

[10] Mundt C.W., Montgomery K.N., Udoh U.E., Barker V.N., Thonier G.C., Tellier A.M., Ricks R.D., Darling R.B., Cagle Y.D., Cabrol N.A. (2005). A multiparameter wearable physiologic monitoring system for space and terrestrial applications. IEEE Trans. Inf. Tech. Biomed..

[11] Miramontes R., Aquino R., Flores A., Rodríguez G., Anguiano R., Ríos A., Edwards A. (2017). PlaIMoS:A remote mobile healthcare platform to monitor cardiovascular and respiratory variables. Sensors.

[12] Zio by iRhythm. https://www.irhythmtech.com/.

[13] Henry I., Bernstein D., Banet M., Mulligan J., Moulton S., Grudic G., Convertino C. Body-Worn, Non-Invasive Sensor for Monitoring Stroke Volume, Cardiac Output and Cardiovascular Reserve. Proceedings of the 2nd Conference on Wireless Health.

[14] Sahoo P.K., Thakkar H.K., Lin W.Y., Chang P.C., Lee M.Y. (2018). On the Design of an Efficient Cardiac Health Monitoring System through Combined Analysis of ECG and SCG Signals. Sensors.

[15] Trung T.Q., Ramasundaram S., Hwang B.U., Lee N.E. (2016). An All-Elastomeric Transparent and Stretchable Temperature Sensor for Body-Attachable Wearable Electronics. Adv. Mater..

[16] Steinhubl S.R., Marriott M.P., Wegerich S.W. (2015). Remote Sensing of Vital Signs: A Wearable, Wireless “Bad-Aid” Sensor with Personalized Analytics for Improved Ebola Patient Care and Worker Safety. Glob. Health Sci. Pract..

[17] Ozemek C., Kirschner M.M., Wilkerson B.S., Byun W., Kaminsky L.A. (2014). Intermonitor reliability of the GT3X+ accelerometer at hip, wrist and ankle sites during activities of daily living. Physiol. Meas..

[18] Bouten C.V.C., Koekkoek K.T.M., Verduin M., Kodde R., Janssen J.D. (1997). A Triaxial Accelerometer and Portable Data Processing Unit for the Assessment of Daily Physical Activity. IEEE Trans. Biomed. Engin..

[19] Ghamari M., Soltanpur C., Cabrera S., Romero R., Martinek R., Nazeran H. Design and Prototyping of a Wristband-Type Wireless Photoplethysmographic Device for Heart Rate Variability Signal Analysis. Proceedings of the 38th Annual International Conference of the IEEE Engineering in Medicine and Biology Society.

[20] Low D.C., Sixon S.J. (2010). Footscan pressure insoles: Accuracy and reliability of force and pressure measurements in running. Gait Posture.

[21] Rodrigues P.J.P. (2013). Sock for Integrated Biometric Monitoring. U.S. Patent.

[22] Ling T.H.Y., Wong L.J., Tan J.E.H., Kiu K.Y. Non-Intrusive Human Body Temperature Acquisition and Monitoring System. Proceedings of the 6th International Conference on Intelligent Systems, Modeling and Simulation.

[23] Sparkfun Body Temperature Sensor. https://www.sparkfun.com/products/8777.

[24] Datasheet of the MCP9700/9700A and MCP9701/9701A Sensors. https://cdn.sparkfun.com/datasheets/E-Textiles/Lilypad/38512_SPCN.pdf.

[25] All about Heart Rate (Pulse) American Heart Association 2015. http://www.heart.org/HEARTORG/Conditions/HighBloodPressure/GettheFactsAboutHighBloodPressure/All-About-Heart-Rate-Pulse_UCM_438850_Article.jsp#.W0DrxfZuJu0.

[26] Sparkfun Pulse Sensor. https://www.sparkfun.com/products/11574.

[27] Datasheet of Sparkfun Pulse Sensor. https://media.digikey.com/pdf/Data%20Sheets/Pulse%20Sensor%20PDFs/Pulse_Sensor.pdf.

[28] Lacuesta R., García L., García-Magariño I., Lloret I. (2017). System to recommend the best place to live based on wellness state of the user employing the heart rate variability. IEEE Access.

[29] Oxygen Saturation Sensor. https://www.sparkfun.com/products/14045?_ga=2.230342172.1255482369.1530462324-1642799772.1528990291.

[30] Datasheet of MAX30105. https://cdn.sparkfun.com/assets/learn_tutorials/5/7/7/MAX30105_3.pdf.

[31] Force Sensitive Resistor. https://www.sparkfun.com/products/9375.

[32] Datasheet of the FSR 0.5” Sparkfun Sensor. https://www.sparkfun.com/datasheets/Sensors/Pressure/fsrguide.pdf.

[33] Wannenburg J., Malekian R. (2017). Physical Activity Recognition from Smartphone Accelerometer Data for User Context Awareness Sensing. IEEE Trans. Syst. Man Cybern. Syst..

[34] ADXL335 Accelerometer. https://www.sparkfun.com/products/9269.

[35] Datasheet of the ADXL335 Accelerometer. https://www.sparkfun.com/datasheets/Components/SMD/adxl335.pdf.

[36] Galvanic Skin Sensor. https://www.seeedstudio.com/Grove-GSR-sensor-p-1614.html.

[37] Specifications of Grove-GSR Sensor. https://www.mouser.com/catalog/specsheets/Seeed_101020052.pdf.

[38] Sendra S., Parra L., Lloret J., Tomás J. (2018). Smart system for children’s chronic illness monitoring. Inf. Fusion.

[39] Wifi Bee ESP V1.0 Module. https://www.amazon.com/LilyPad-Arduino-Module-Wireless-Project/dp/B01LVZ4CC3.

[40] Lavery L.A., Agrawal C.M., Athanaslou K.A., Constantinides G.P., Lanctot D.R., Zamorano R.G. (2004). Foot Temperature and Health Monitoring System. U.S. Patent.

[41] Monnard C.R., Fares E., Calonne J., Miles-Chan J.L., Montani J., Durrer D., Schutz Y., Dullo G. (2017). Issues in Continuous 24-h Core Body Temperature Monitoring in Humand Using an Ingestible Capsule Telemetric Sensor. Front. Endocrinol..

[42] Wu K., Zhang Y. Contactless and Continuous Monitoring of Heart Electric Activities through Clothes on a Sleeping Bed. Proceedings of the 5th International Conference on Information Technology and Application in Biomedicine.

[43] Sendra S., Lloret J., García M., Toledo J.F. (2011). Power saving and energy optimization techniques for Wireless Sensor Networks. J. Commun..

[44] Heinzelman W.R., Chandrakasan A., Balakrishnan H. Energy-Efficient Communication Protocol for Wireless Microsensor Networks. Proceedings of the IEEE 33rd Annual Hawaii International Conference on System Sciences.

[45] Mohsen N.A., Mozaffari-Kermani M., Sur-Kolay S., Raghunathan A., Jha N.K. (2015). Energy-Efficient Long-term Continuous Personal Health Monitoring. IEEE Trans. Multi-Scale Comp. Syst..

